# Supplementary material for: Variational inference for microbiome survey data with application to global ocean data
Source: ISME Commun. 2025 May 2;5(1):ycaf062. doi: 10.1093/ismeco/ycaf062 (PMC12064564; doi:10.1093/ismeco/ycaf062)
Supplement: vi-midas_supp_ycaf062 [file vi-midas_supp_ycaf062.pdf]

Variational inference for microbiome survey data with application to global ocean data

Aditya Mishra[1*], Jesse McNichol[3,4], Jed Fuhrman[4], David Blei[2,5], and Christian L. Müller[2,6,7]

[1]Department of Statistics, University of Georgia, Athens, GA, USA
[2]Center for Computational Mathematics, Flatiron Institute, New York, NY, USA
[3]Department of Biology, St. Francis Xavier University, Antigonish, NS, Canada
[4]Department of Biological Sciences, University of Southern California, Los Angeles
[5]Department of Statistics and Computer Science, Columbia University, New York
[6]Computational Health Center, Helmholtz Zentrum München, Germany
[7]Department of Statistics, LMU München, Germany

April 7, 2025

1 Supplementary Figures

We report the supplementary figures of the data analysis related to the main manuscript.

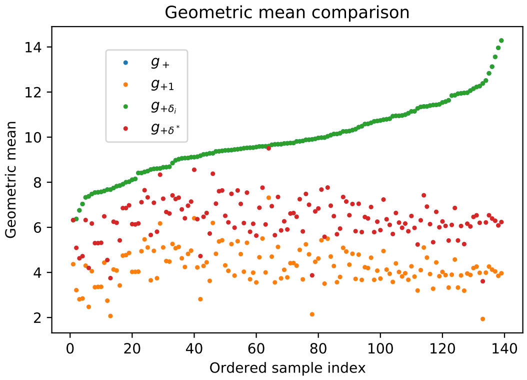

(a)

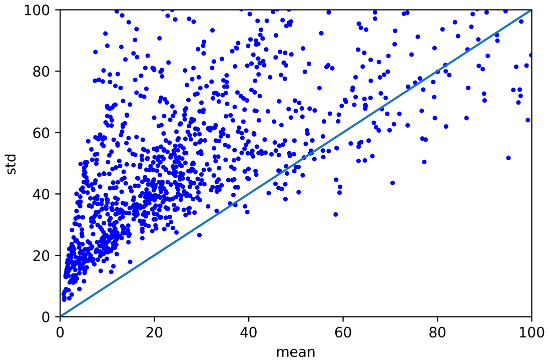

(b)

Figure S2: a) Compares the possible approach of computing the geometric mean of the microbial abundance sample to be considered as an offset term $\mathbf{t}$ in the VI-MIDAS. Suggested approach mainly differs in the pseudo δ_i added to zero entries in a given sample; b) Over-dispersion of abundance is demonstrated by comparing the means and standard deviations of the abundance of $q = 1378$ species selected for analysis. According to the analysis, the negative binomial distribution is an appropriate distribution to represent the microbial abundance data.

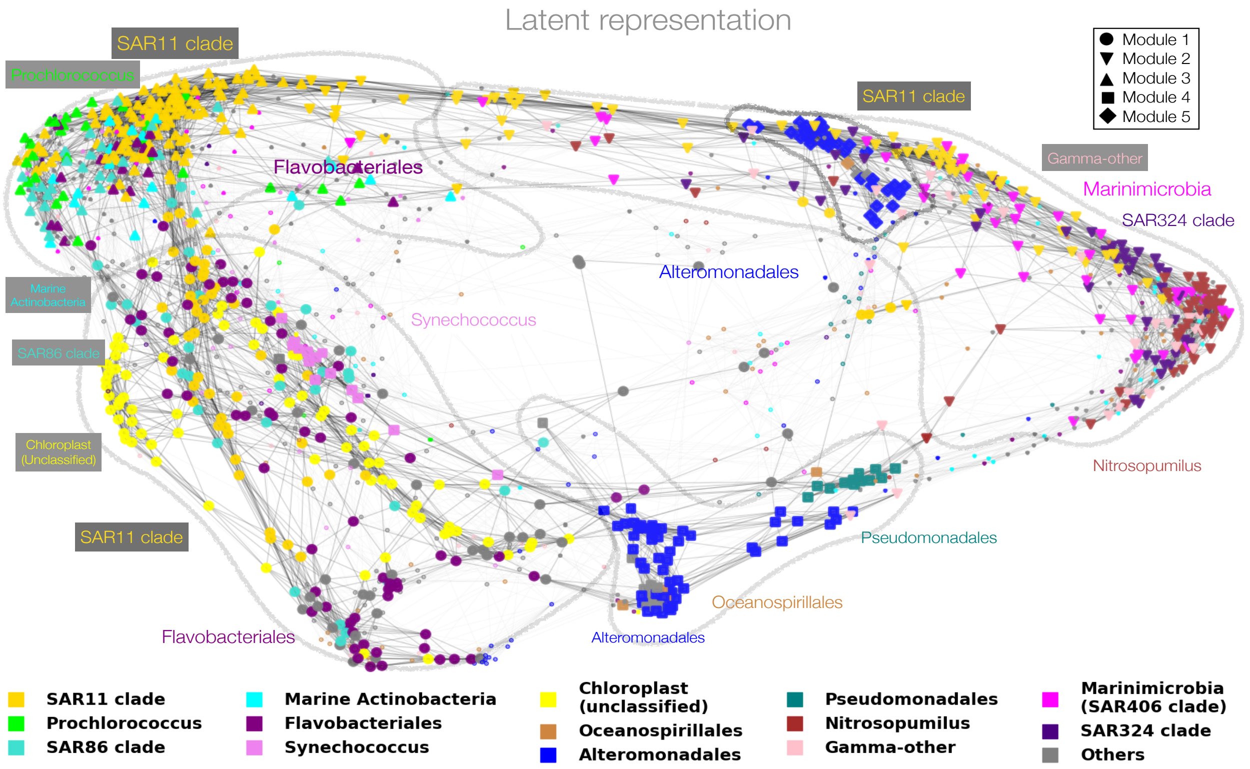

Figure S1: Low-dimensional embedding of the latent representation $\boldsymbol{\beta}$ using a k-nearest-neighbor ($k_{\mathrm{nn}} = 10$) graph of cosine distances. Modularity analysis reveals five distinct graph modules. We highlight 825 out of a total of 1378 taxa, comprising the top five ERCs (color-coded) in each of the five modules (see main text for further information).

2 Supplementary Tables

Table S1: Dimension of the latent variables in the parameter set $\boldsymbol{\ell}$ of the VI-MIDAS model.

	$\boldsymbol{\gamma}$	$\boldsymbol{\beta}$	$\boldsymbol{\rho}$	$\boldsymbol{\alpha}$	$\boldsymbol{\delta}$	$\boldsymbol{\vartheta}$	$\boldsymbol{\tau}$	$\boldsymbol{\Phi}$
# ncols	1378	1378	1378	4	4	4	1378	1378
# nrows	11	200	200	200	200	200	1	1

Table S2: Selected value of the latent variable dimension k and the hyper-parameters $\{\lambda, \upsilon\}$ of the Laplace and Inverse-Cauchy priors in the range of one standard deviation of the largest value of the LLPD_o.

Index	k	λ	υ	LLPD_o
1	200	0.246	0.10063	-3.318726

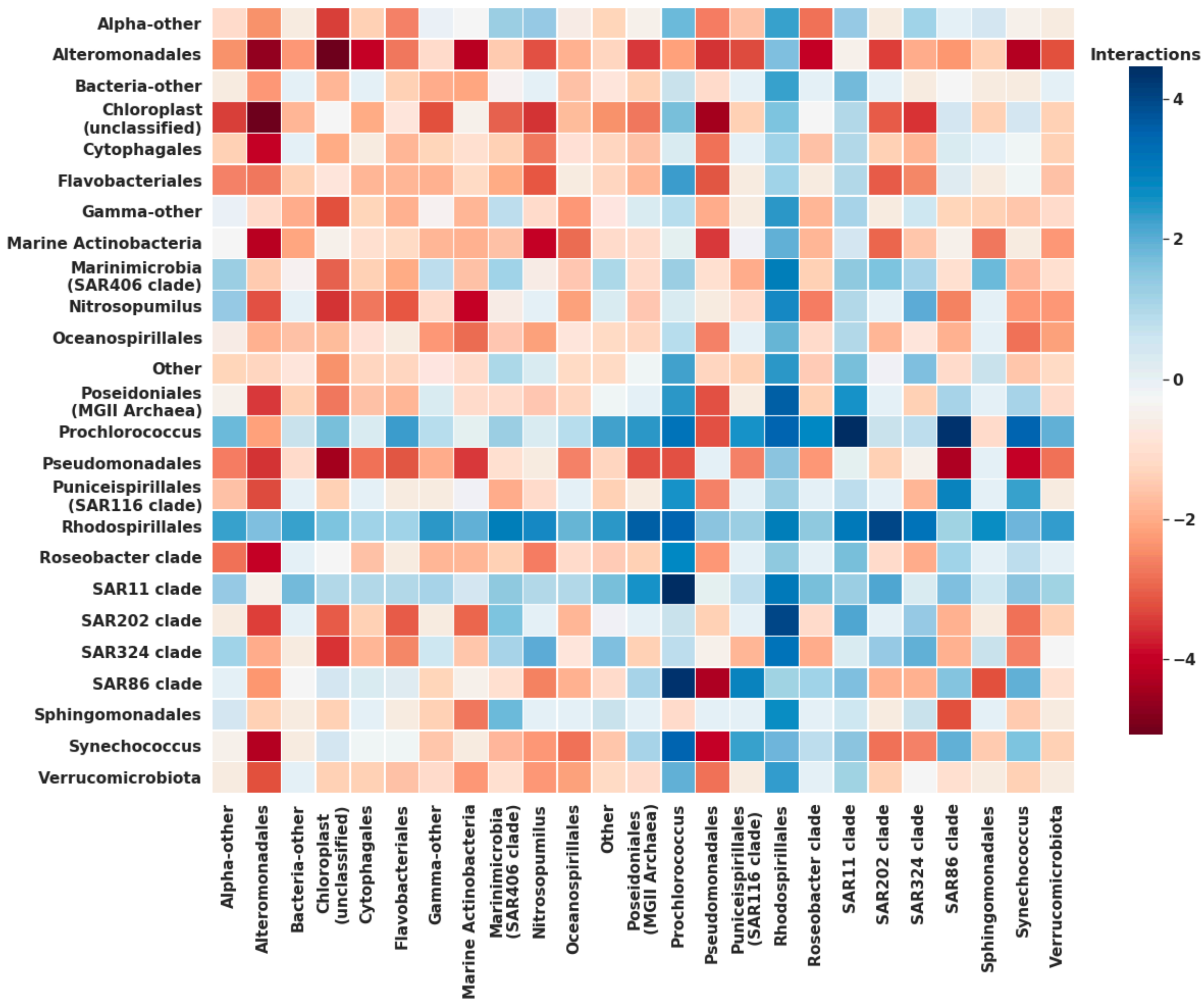

Figure S3: Comparison of interactions among ERC identifiers: The adjacency matrices of significant mutualistic and competitive interactions among OTUs are grouped and aggregated by their ERC identifier. A block represents the logarithmic ratio of the number of mutualistic interactions versus the number of competitive interactions. Positive and negative values, accordingly, represent one larger than the other. Blue for mutualistic interaction and Red for competitive interactions.

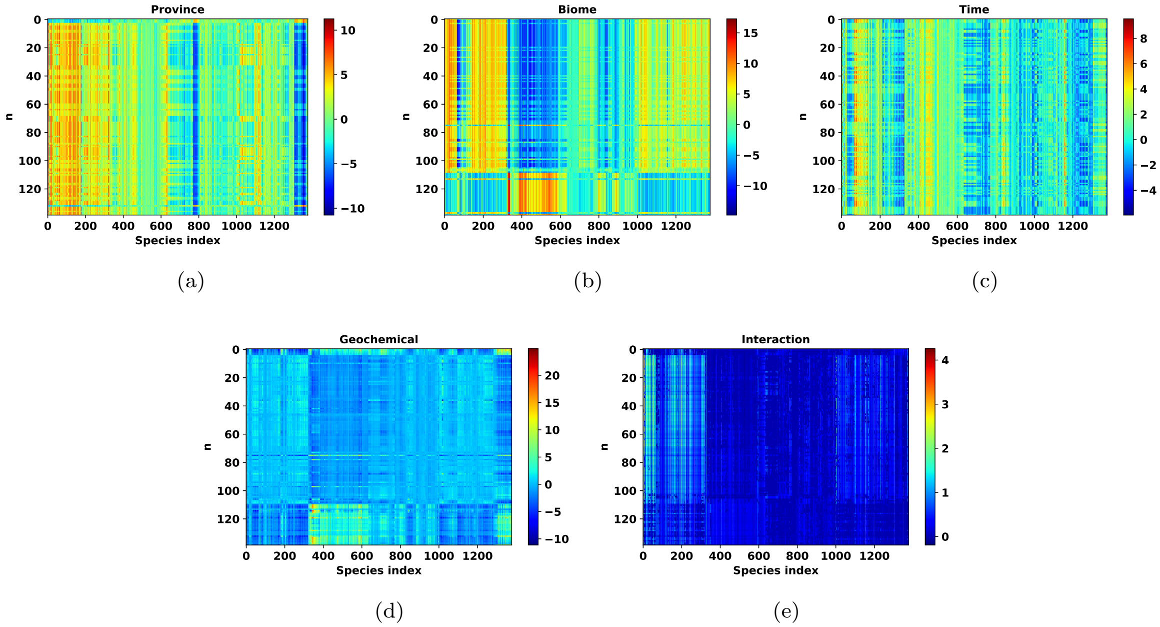

Figure S4: Model fit: Estimate of the non-interaction components in the linear predictor $\boldsymbol{\eta}$: a) Province indicator; b) Biome (depth) indicator; a) Quarter (time) indicator; d) Biogeochemical factor; e) Embedding effect.

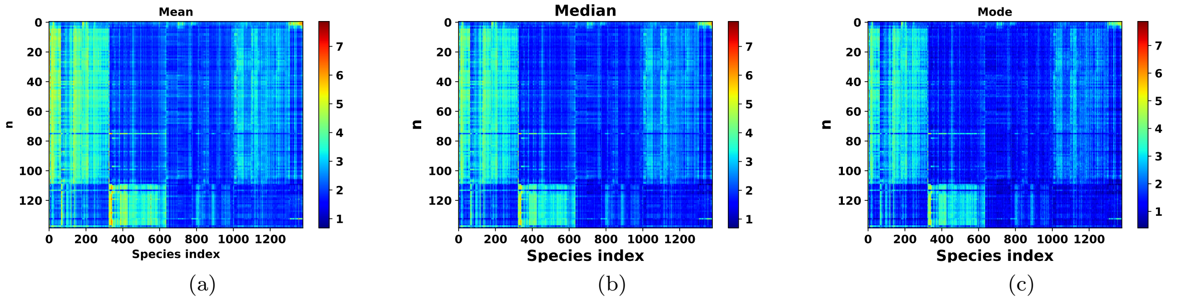

Figure S5: Model fit: a) Mean estimate of the posterior sample using VI-MIDAS; b) Median estimate of the posterior sample using VI-MIDAS; a) Mode estimate of the posterior sample using VI-MIDAS.

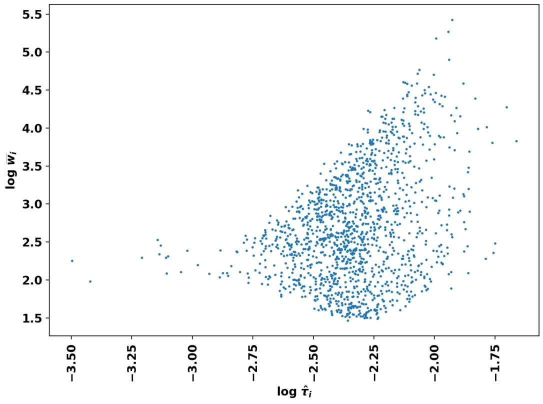

(a)

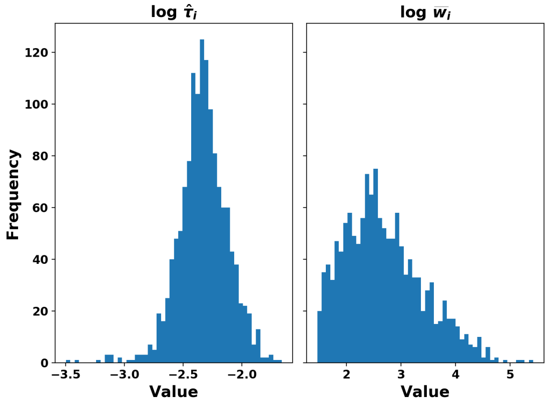

(b)

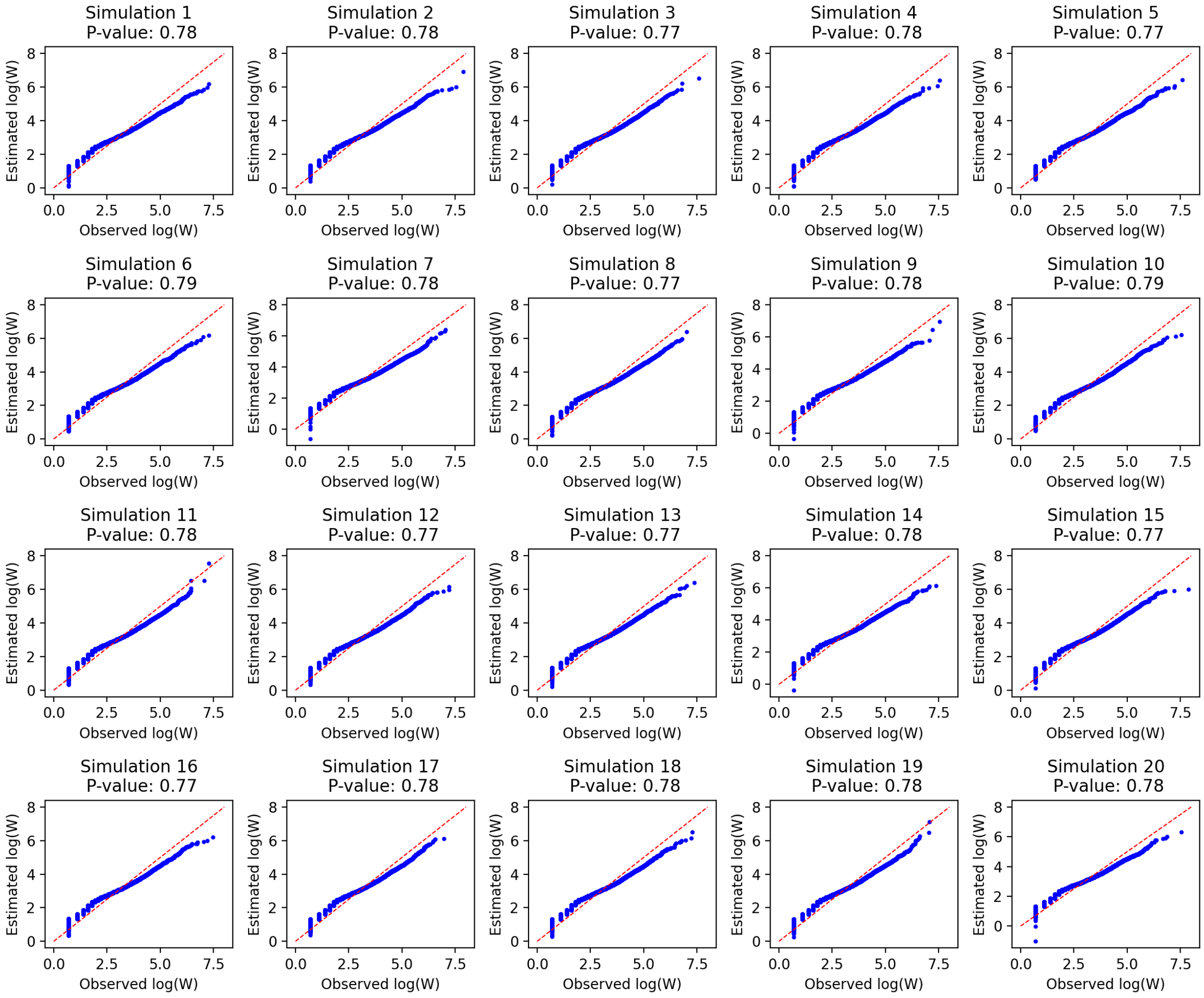

(c)

Figure S6: a) Scatter plot comparing entries of the CLR transform of the observed and estimated microbial species abundance given by $\log \mathbf{W}/\mathbf{T}$ and $\log \mathrm{E}(\mathbf{W})/\mathbf{T}$, respectively; b) Comparison of the estimate of τ and mean of the species abundance; and c) Out-of-sample validation of VI-MIDAS model performance using Q-Q plots comparing predicted vs. observed values for 20 simulations with 90% training and 10% validation data. Each panel represents one simulation and reports p-values from Bayesian model tests for significant differences between predicted and observed data across the 20 simulations.

Table S3: Top five ecologically relevant classifications (ERC) indicator of taxa in the five modules identified in the network (shown in Figure 5) highlighting similar nodes. The table reports the composition (as %) of 825 taxa in terms of the ERC indicator and their mean abundance.

Module	ERC	Abundance (Mean)	Members (%)	Module	ERC	Abundance (Mean)	Members (%)
	Chloroplast	12.49	9.09		Alteromonadales	7.24	6.42
	Flavobacteriales	11.00	8.00		Synechococcus	27.70	1.70
1	SAR11 clade	19.44	5.94	4	Pseudomonadales	4.83	1.45
	Other	10.77	5.09		Other	6.37	1.33
	SAR86 clade	18.91	3.88		Oceanospirillales	5.78	0.48
	SAR11 clade	18.67	8.48		Alteromonadales	17.84	3.76
	Nitrosopumilus	23.03	6.06		Bacteria-other	12.22	0.12
2	SAR324 clade	15.48	5.09	5	Chloroplast	17.81	0.12
	Marinimicrobia	16.69	4.97		Oceanospirillales	36.30	0.12
	Gamma-other	11.13	3.15		Other	13.29	0.12
	SAR11 clade	45.63	13.70				
	Prochlorococcus	53.06	3.52				
3	SAR86 clade	34.74	3.27				
	Flavobacteriales	28.33	2.06				
	Marine Actinobacteria	29.17	2.06				

3 Methods Details

3.1 Controlling for the relative abundance data in VI-MIDAS

Due to experimental limitations and systematic bias in high-throughput sequencing, we do not have access to microbial species' absolute (actual) abundance data. Several analysis techniques have been proposed for use on the transformed data. Some acceptable transformation techniques include centered log-ratio transform (CLR), relative abundance, and isometric log-ratio transform [Callahan et al., 2016]. Many statistical methods [Shi et al., 2016, Mishra et al., 2019] consider the relative abundance (compositional) data for exploratory and predictive tasks in microbiome data analysis. For example, CLR transformation leads to centering the log of abundance by its geometric mean, i.e.,

$$\mathrm{CLR}(\mathbf{w}) = \log \mathbf{w} - \log g(\mathbf{w})$$

where $g(\mathbf{w})$ is the geometric mean of $\mathbf{w}$. Instead of using the relative abundance data for a predictive model, VI-MIDAS overcomes the limitations of the microbial abundance data by the use of a suitable offset term. By including $t_i = \log g(\mathbf{w}_i)$ as an offset term in the log-link function (2) of VI-MIDAS, one can control for such limitations; see [Zhang et al., 2017].

A significant number of entries in the abundance matrix $\mathbf{W}$ are zeros. Some of these may reflect the absence of species, while others may be caused by experimental limitations. Hence, one may consider computing the geometric mean using only non-zero entries in a sample (g_+) or after adding a pseudocount (δ_i) to the ith sample. A standard approach is to use $\delta_i = 1$, denoted g_{+1}. In the VI-MIDAS model, we mainly follow de la Cruz and Kreft [2018] to compute the pseudocount δ_i by solving

$$\delta_i = \sup\{\delta^* \in (0, \infty) \mid G_{\mathbf{w}_i,\epsilon}(\mathbf{w}_i) - g(\mathbf{w}_i^+) \leq \epsilon g(\mathbf{w}_i^+)\},$$

where $G_{\mathbf{w}_i,\epsilon}(\mathbf{w}_i) = \exp\left(\frac{1}{n}\sum_{j=1}^{q} \log(w_{ij} + \delta_i)\right) - \delta_i$, $\mathbf{w}_i^+ = \{w_{ij} | w_{ij} > 0\}$ is the set of nonzero entries in $\mathbf{w}_i$ and ϵ is the relative difference between the standard geometric mean $g(\mathbf{w}_i^+)$ and our modified geometric mean. Let us denote the geometric mean by $g_{+\delta_i}$. In practice, one may also use $\delta^* = \min_{i=1}^{n} \delta_i$ as a possible alternatives with geometric mean as $g_{+\delta^*}$. Figure S2 (a) in the supplementary materials compares the geometric mean of n samples using the suggested approach.

3.2 Variational inference for estimation

VI-MIDAS is an over-parameterized model. To generalize well on the test data, VI-MIDAS uniformly places a Laplace prior with scale parameter λ on each of the unconstrained latent variables in the set $\{\boldsymbol{\alpha}, \boldsymbol{\delta}, \boldsymbol{\beta}, \boldsymbol{\gamma}, \boldsymbol{\rho}, \boldsymbol{\vartheta}\}$, i.e., $p(\alpha_{ij}) = \text{Laplace}(0, \lambda)$ and $p(\boldsymbol{\alpha}) = \prod_{i,j} p(\alpha_{ij})$. Also, we place a inverse-Cauchy priors on the dispersion parameter $\boldsymbol{\Phi}$, i.e., $p(\phi_j) = \text{inverse-Cauchy}(0, \upsilon)$ and $p(\boldsymbol{\Phi}) = \prod_j p(\phi_j)$, and a Uniform(1,2) prior for the shape parameter $\boldsymbol{\tau}$, i.e., $\tau_j \sim \text{Beta(1,1)}$ and $p(\boldsymbol{\tau}) = \prod_j p(\tau_j)$. Given the microbial abundance data $\mathbf{W}$, the geochemical covariates $\mathbf{X}$, the model parameter $\boldsymbol{\ell}$ and the generative model (1), we express the posterior as

$$p(\boldsymbol{\ell}; \mathbf{W}, \mathbf{X}, \mathbf{t}) = \frac{p(\mathbf{W}; \boldsymbol{\ell}, \mathbf{X}, \mathbf{t}) p(\boldsymbol{\ell})}{p(\mathbf{W}; \mathbf{X}, \mathbf{t})}, \tag{1}$$

where $p(\mathbf{W}; \boldsymbol{\ell}, \mathbf{X}, \mathbf{t}) = \prod_{i,j} p(w_{ij}; \tau_j \mu_{ij}, \phi_j)$ is the likelihood of $\mathbf{W}$ and $p(\boldsymbol{\ell}) = p(\boldsymbol{\alpha})p(\boldsymbol{\delta})p(\boldsymbol{\beta})p(\boldsymbol{\gamma})p(\boldsymbol{\rho})p(\boldsymbol{\Phi})p(\boldsymbol{\tau})p(\boldsymbol{\vartheta})$ is the joint prior distribution. In the high-dimensional setting, computing the posterior distribution is challenging because of the intractable form of the marginal distribution $p(\mathbf{W}; \mathbf{X}, \mathbf{t})$ and the non-conjugate priors on the model parameters. Markov Chain Monte Carlo (MCMC) sampling provides a helpful paradigm for obtaining the required posterior distribution in the Bayesian framework. However, MCMC lacks computational efficiency in large/high-dimensional problems such as VI-MIDAS. Hence, we use the framework of mean-field VI [Jordan et al., 1999, Wainwright et al., 2008, Blei et al., 2017] and approximate the posterior with a variational posterior distribution of the latent variable $\boldsymbol{\ell}$.

We let $q(\boldsymbol{\ell}; \boldsymbol{\nu})$ be the variational posterior distribution with parameter $\boldsymbol{\nu}$. VI minimizes the Kullback-Leibler (KL) divergence,

$$\min_{\boldsymbol{\nu}} \ \text{KL}(q(\boldsymbol{\ell}; \boldsymbol{\nu}) \,||\, p(\boldsymbol{\ell}; \mathbf{W}, \mathbf{X}, \mathbf{t}))$$

such that $\text{supp}(q(\boldsymbol{\ell}; \boldsymbol{\nu})) \subseteq \text{supp}(p(\boldsymbol{\ell}; \mathbf{W}, \mathbf{X}, \mathbf{t}))$. On simplification, the optimization problem is equivalent to maximizing the evidence lower bound (ELBO) given by

$$\mathcal{L}(\boldsymbol{\nu}) = \mathbb{E}_{q(\boldsymbol{\ell};\boldsymbol{\nu})}[\log P(\mathbf{W}, \boldsymbol{\ell}; \mathbf{X}, \mathbf{t})] - \mathbb{E}_{q(\boldsymbol{\ell};\boldsymbol{\nu})}[\log q(\boldsymbol{\ell}; \boldsymbol{\nu})], \tag{2}$$

a lower bound on the logarithm of the joint probability of the observations, i.e., $\log P(\mathbf{W}; \mathbf{X}, \mathbf{t})$ [Jordan et al., 1999]. One can further simplify by replacing the joint distribution $P(\mathbf{W}, \boldsymbol{\ell}; \mathbf{X}, \mathbf{t})$ with a product of likelihood and prior distribution $P(\mathbf{W}, \boldsymbol{\ell}; \mathbf{X}, \mathbf{t}) = P(\mathbf{W}; \boldsymbol{\ell}, \mathbf{X}, \mathbf{t}) P(\boldsymbol{\ell})$.

Coordinate ascent variational inference provides an efficient framework for approximating the variational posterior in a generative model with conjugate priors satisfying support-matching constraints [Blei et al., 2017]. For a general scenario, such as VI-MIDAS, we transform the support of the latent variable $\boldsymbol{\ell}$ to a real coordinate space using a one-to-one differentiable function

$$\mathbf{T} : \text{supp}(p(\boldsymbol{\ell})) = \mathbb{R}^l \tag{3}$$

and express the transformed variable as $\boldsymbol{\zeta} = \mathbf{T}(\boldsymbol{\ell})$ where $\boldsymbol{\zeta} \in \mathbb{R}^l$. For example, given any latent variable $a \in \mathbb{R}^+$, then for $T = \log$, we have $\zeta = T(a) \in \mathbb{R}$. Similarly, for any $a \in (0, 1)$, we apply the logit transform and write $\zeta = T(a) = \text{logit}(a) = \log(\frac{a}{1-a}) \in \mathbb{R}$. Using a standard transformation, we express the joint distribution of the transformed latent variable $\boldsymbol{\zeta}$ as $P(\mathbf{W}, \mathbf{T}^{-1}(\boldsymbol{\zeta}); \mathbf{X}, \mathbf{t}) |\det \mathbf{J}_{\mathbf{T}^{-1}}(\boldsymbol{\zeta})|$. For the unconstrained latent variables $\boldsymbol{\zeta}$, we formulate the mean-field variational posterior distribution as

$$q(\boldsymbol{\zeta}; \boldsymbol{\mu}, \boldsymbol{\sigma}) \sim \mathcal{N}(\boldsymbol{\mu}, \boldsymbol{\Sigma}) = \prod_{i=1}^{l} \mathcal{N}(\zeta_i; \mu_i, \sigma_i),$$

where $\boldsymbol{\mu} = [\mu_1, \ldots, \mu_l] \in \mathbb{R}^l$ is the mean parameter and $\boldsymbol{\sigma} = [\sigma_1, \ldots, \sigma_l] \in \mathbb{R}^{+l}$ is the variance parameter. Now, we reformulate the ELBO (2) in terms $\boldsymbol{\zeta}$ as

$$\begin{aligned} \mathcal{L}(\boldsymbol{\mu}, \boldsymbol{\sigma}) &= \mathbb{E}_{q(\boldsymbol{\zeta};\boldsymbol{\mu},\boldsymbol{\sigma})}[\log P(\mathbf{W}, \mathbf{T}^{-1}(\boldsymbol{\zeta}); \mathbf{X}, \mathbf{t}) + \log |\det \mathbf{J}_{\mathbf{T}^{-1}}(\boldsymbol{\zeta})|] - \mathbb{E}_{q(\boldsymbol{\zeta};\boldsymbol{\mu},\boldsymbol{\sigma})}[\log q(\boldsymbol{\zeta}; \boldsymbol{\kappa})] \\ &= \mathbb{E}_{q(\boldsymbol{\zeta};\boldsymbol{\mu},\boldsymbol{\sigma})}[\log P(\mathbf{W}, \mathbf{T}^{-1}(\boldsymbol{\zeta}); \mathbf{X}, \mathbf{t}) + \log |\det \mathbf{J}_{\mathbf{T}^{-1}}(\boldsymbol{\zeta})|] + \sum_i \log \sigma_i + const. \end{aligned} \tag{4}$$

Here, Gaussian variational distribution is related to the second-order approximation of the posterior around the maximum-a-posteriori (MAP) estimate. In terms of the original parameter $\boldsymbol{\ell}$, variational distribution is non-Gaussian because of the transform $\mathbf{T}$ and its Jacobian. To estimate parameters of the variational posterior after reparameterization, VI-MIDAS solves

$$\widehat{\boldsymbol{\mu}},\ \widehat{\boldsymbol{\sigma}} \equiv \arg\max_{\boldsymbol{\mu},\boldsymbol{\sigma}} \mathcal{L}(\boldsymbol{\mu},\boldsymbol{\sigma}), \tag{5}$$

using the coordinate ascent approach. We solve the optimization problem using stochastic gradient ascent (SGA), which uses automatic differentiation (AD) to compute the gradient and Monte Carlo integration to approximate the expectation [Blei et al., 2017]. AD is applicable when gradient operation is inside the expectation. The estimation approach achieves this by applying an additional elliptical transformation given by $\kappa_i = (\zeta_i - \mu_i)/\exp(v_i)$ where $v_i = \log\sigma_i$. We denote the set of new latent variable as $\boldsymbol{\kappa} = [\kappa_1,\ldots,\kappa_l]$, and reparameterize ELBO as

$$\mathcal{L}(\boldsymbol{\mu},\mathbf{v}) = \mathbb{E}_{q(\boldsymbol{\kappa};\mathbf{0},\mathbf{1})}[\log P(\mathbf{W},\mathbf{T}^{-1}(\mathbf{S}(\boldsymbol{\kappa}));\mathbf{X},\mathbf{t}) + \log|\det \mathbf{J}_{\mathbf{T}^{-1}}(\mathbf{S}(\boldsymbol{\kappa}))|] + \sum_i v_i, \tag{6}$$

where $\boldsymbol{\zeta} = \mathbf{S}(\boldsymbol{\kappa}) = \mathrm{diag}[exp(\mathbf{v})]\boldsymbol{\kappa} + \boldsymbol{\mu}$ and $\mathbf{v} = [v_1,\ldots,v_l]$. To execute gradient ascent, we need to compute $\frac{d\mathcal{L}(\boldsymbol{\mu},\mathbf{v})}{d\boldsymbol{\mu}}$ and $\frac{d\mathcal{L}(\boldsymbol{\mu},\mathbf{v})}{d\mathbf{v}}$. Let us represent the derivative of the random variable function inside the expectation as $\frac{da}{d\boldsymbol{\kappa}} = \nabla_{\boldsymbol{\theta}}\log P(\mathbf{W},\boldsymbol{\theta};\mathbf{X},\mathbf{t})\nabla_{\boldsymbol{\kappa}}\mathbf{T}^{-1}(\mathbf{S}(\boldsymbol{\kappa})) + \nabla_{\boldsymbol{\kappa}}\log|\det \mathbf{J}_{\mathbf{T}^{-1}}(\mathbf{S}(\boldsymbol{\kappa}))|$. Then, with reparameterized ELBO, the gradient with respect to $\boldsymbol{\mu}$ and $\mathbf{v}$ is given by

$$\frac{d\mathcal{L}(\boldsymbol{\mu},\mathbf{v})}{d\boldsymbol{\mu}} = \mathbb{E}_{q(\boldsymbol{\kappa};\mathbf{0},\mathbf{1})}\left[\frac{da}{d\boldsymbol{\kappa}}\right] \quad \text{and} \quad \frac{d\mathcal{L}(\boldsymbol{\mu},\mathbf{v})}{d\mathbf{v}} = \mathbb{E}_{q(\boldsymbol{\kappa};\mathbf{0},\mathbf{1})}\left[\frac{da}{d\boldsymbol{\kappa}} \odot exp(\mathbf{v}) \odot \boldsymbol{\kappa}\right] + \mathbf{1}. \tag{7}$$

We compute the gradient inside the expectation using automatic differentiation and then approximate the expectation using MC integration by drawing m (typically $m = 1$) from the standard normal distribution. This step results in a noisy and unbiased estimate of the gradient of the ELBO. Using the gradient estimate, we update the variational parameters $\{\boldsymbol{\mu},\mathbf{v}\}$ via stochastic optimization given by

$$\boldsymbol{\mu}^{(i+1)} \leftarrow \boldsymbol{\mu}^{(i)} + \boldsymbol{\xi}_{\boldsymbol{\mu}}^{(i)} \odot \frac{d\mathcal{L}(\boldsymbol{\mu},\mathbf{v})}{d\boldsymbol{\mu}}, \qquad \text{and} \qquad \mathbf{v}^{(i+1)} \leftarrow \mathbf{v}^{(i)} + \boldsymbol{\xi}_{\mathbf{v}}^{(i)} \odot \frac{d\mathcal{L}(\boldsymbol{\mu},\mathbf{v})}{d\mathbf{v}}$$

where $\boldsymbol{\xi}_{\boldsymbol{\mu}}^{(i)}$ and $\boldsymbol{\xi}_{\mathbf{v}}^{(i)}$ are step-size. The computation procedure is guranteed to converge to a local maximum of the ELBO under the condition of a sufficiently decaying step-size sequence. In particular, we use an adaptive step-size sequence [Duchi et al., 2011] to meet this condition given by

$$\boldsymbol{\xi}_{\boldsymbol{\mu}}^{(i)} = \vartheta \times i^{-1/2+\varepsilon} \times \left(\varsigma + \sqrt{\boldsymbol{\varrho}_{\boldsymbol{\mu}}^{(i)}}\right)^{-1} \quad \text{and} \quad \boldsymbol{\xi}_{\mathbf{v}}^{(i)} = \vartheta \times i^{-1/2+\varepsilon} \times \left(\varsigma + \sqrt{\boldsymbol{\varrho}_{\mathbf{v}}^{(i)}}\right)^{-1}, \tag{8}$$

where ϑ is the learning rate, $\boldsymbol{\varrho}_{\boldsymbol{\mu}}^{(i)} = \alpha\frac{d\mathcal{L}(\boldsymbol{\mu},\mathbf{v})}{d\boldsymbol{\mu}} + (1-\alpha)\boldsymbol{\varrho}_{\boldsymbol{\mu}}^{(i-1)}$ and $\boldsymbol{\varrho}_{\mathbf{v}}^{(i)} = \alpha\frac{d\mathcal{L}(\boldsymbol{\mu},\mathbf{v})}{d\mathbf{v}} + (1-\alpha)\boldsymbol{\varrho}_{\mathbf{v}}^{(i-1)}$ is the curvature. In practice, we search for the optimal learning rate such that $\vartheta \in \{0.01, 0.1, 1, 10, 100\}$. Other parameters, such as $\varepsilon = 10^{-16}$, $\varsigma = 1$ are chosen to prevent zero division, and $\alpha = 0.1$ are chosen in order to give more weight to the past curvature. This suggested approach to parameter estimation comes under the framework of Automatic Differentiation Variational Inference (ADVI) [Kucukelbir et al., 2017]. The approach is implemented in the probabilistic programming language Stan [Carpenter et al., 2017]. We have summarized the parameter estimation procedure in Algorithm 1.

Algorithm 1 Automatic Differentiation Variational Inference for VI-MIDAS

Given: Count abundance data $\mathbf{W}$, covariates $\mathbf{X}$, province $\mathbf{P}$, ocean depth $\mathbf{D}$, model $P(\mathbf{W}, \mathbf{T}^{-1}(\mathbf{S}(\boldsymbol{\kappa})); \mathbf{X}, \mathbf{t})$ where $\mathbf{S}(\boldsymbol{\kappa}) = \text{diag}[exp(\mathbf{v})]\boldsymbol{\kappa} + \boldsymbol{\mu}$.

Initialize variational posterior parameter $\boldsymbol{\mu}^{(1)} = \mathbf{0}, \mathbf{v}^{(1)} = \mathbf{0}$, $i = 1$.

Search for learning parameter rate ϑ over a set of finite values.

repeat

Draw $m = 1$ sample from $\mathcal{N}(\mathbf{0}, \mathbf{I})$.

Estimate noisy gradient $\frac{d\mathcal{L}(\boldsymbol{\mu},\mathbf{v})}{d\boldsymbol{\mu}}$ and $\frac{d\mathcal{L}(\boldsymbol{\mu},\mathbf{v})}{d\mathbf{v}}$ using MC integration; see equation (7).

Calculate step-size $\boldsymbol{\xi}_{\mu}^{(i)}$ and $\boldsymbol{\xi}_{\mathbf{v}}^{(i)}$ using ADAM; see equation (8).

Update $\boldsymbol{\mu}^{(i+1)} \leftarrow \boldsymbol{\mu}^{(i)} + \boldsymbol{\xi}_{\mu}^{(i)} \odot \frac{d\mathcal{L}(\boldsymbol{\mu},\mathbf{v})}{d\boldsymbol{\mu}}$ and $\mathbf{v}^{(i+1)} \leftarrow \mathbf{v}^{(i)} + \boldsymbol{\xi}_{\mathbf{v}}^{(i)} \odot \frac{d\mathcal{L}(\boldsymbol{\mu},\mathbf{v})}{d\mathbf{v}}$.

$i \leftarrow i + 1$

until $|\mathcal{L}(\boldsymbol{\mu}^{(i+1)}, \mathbf{v}^{(i+1)}) - \mathcal{L}(\boldsymbol{\mu}^{(i)}, \mathbf{v}^{(i)})| < \epsilon$ where $\epsilon = 0.01$

return $\boldsymbol{\mu}^* = \boldsymbol{\mu}^{(i)}, \mathbf{v}^* = \mathbf{v}^{(i)}$.

3.3 Hyperparameter tuning

We estimate the model parameters using the ADVI approach implemented in the probabilistic programming language Stan. VI-MIDAS requires specifying the dimension k of the latent variable and the hyperparameters $\{\lambda, \upsilon\}$ of the sparsity-inducing Laplace priors and the inverse-Cauchy prior of the generative model. In the hyperparameter tuning step, the procedure selects 50 random settings from a range of possible values of the parameters given by $k \in \{10, 16, 30, 50, 80, 100, 150, 200, 500\}$, $\lambda \in (0.01, 3000)$ and $\upsilon \in (0.03125, 0.5)$. To evaluate these settings, we split the data into five folds with 90% training and 10% testing. We use Algorithm 1 to estimate parameters on the former and then evaluate the estimate on the latter using out-of-sample log pointwise predictive density (LLPD) [Gelman et al., 2013, Blei et al., 2017]. Figure S7 reports the test sample LLPD to evaluate the model fit for the selected settings. We select all the settings in the range of one standard deviation of the most significant value of the LLPD; see Table S2 in the supplementary materials.

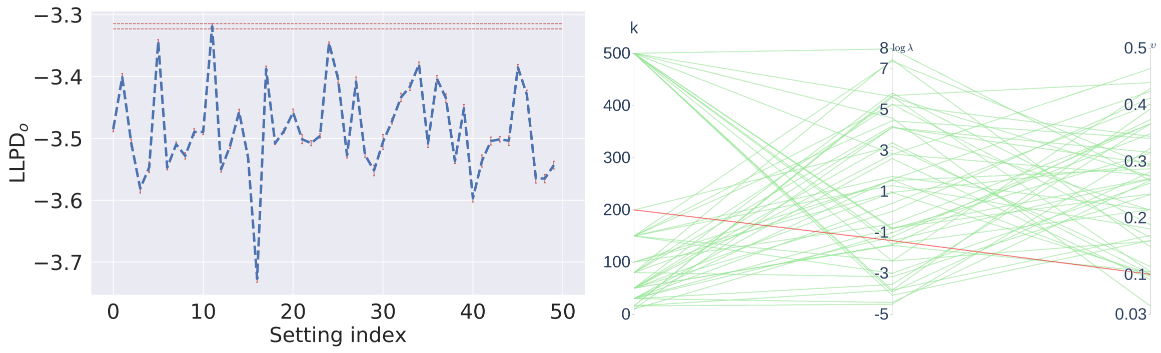

(a) (b)

Figure S7: Hyperparameter tuning: a) out-of-sample log pointwise predictive density (LLPD) comparing fifty different settings randomly selected from a range possible values: taxa-specific feature vector length $k \in \{10, 16, 30, 50, 80, 100, 150, 200, 500\}$, hyperparameter for Laplace priors $\lambda \in (0.01, 3000)$ and the hyperparameter for inverse-Cauchy priors $\upsilon \in (0.03125, 0.5)$; b) parallel coordinate plot highlighting the hyperparameter settings of high (H : dark red) and low (L: light green) mean values of LLPD. The analysis has selected $k = 200$, $\lambda = 0.246$ and $\upsilon = 0.10063$ as the values of the latent variable dimension and the hyperparameters with the highest LLPD $= -3.32$.

3.4 Ablation Study

The analysis quantifies the relative importance of each of the components, i.e., interaction(-I), biogeochemical environment(-E), province(-P), ocean depth(-D), and seasonality(-S) in the VI-MIDAS model. For each component in the VI-MIDAS model, we consider a component-excluded model. For instance, the model that excludes the interaction component is denoted VI-MIDAS(-I). Similarly, VI-MIDAS(-E), VI-MIDAS(-P), VI-MIDAS(-D) and VI-MIDAS(-S) denote the models excluding the geochemical environment(E), province(P), ocean depth(D) and seasonality(S) components, respectively. For the selected setting from the hyperparameter tuning step, we evaluate each component-excluded model in terms of the out-of-sample LLPD. From the comparison results in Table S4, we observe that the seasonality (S) is least important and the ocean depth(D) component is most important.

Table S4: Out-of-sample log-likelihood posterior predictive density (LLPD) of the full model and after ablation of the environmental(E), province(P), ocean depth(D), seasonality(S), and latent interaction (I) component.

**Model**	VI-MIDAS	VI-MIDAS(-E)	VI-MIDAS(-P)	VI-MIDAS(-D)	VI-MIDAS(-S)	VI-MIDAS(-I)
**LLPD**	-3.3**322**	-3.3**554**	-3.3**398**	-3.3**882**	-3.3**335**	-3.3**377**

3.5 Model sensitivity analysis

The objective function maximizing the ELBO is non-convex; hence, the estimates of the parameters are sensitive to their initial values. For each of the settings selected in Table S2, we estimate the model parameters on complete data for fifty random initializations. The parameters estimated from the random initializations are evaluated based on the fitted LLPD [Gelman et al., 2013]. Out of fifty different parameter estimates for the selected setting, we select the one with the most significant value of fitted LLPD. Based on the variational posterior parameter estimate, we generate 100 posterior samples of the latent variables $\boldsymbol{\ell}$.

3.6 Model fit diagnostic

After performing the model sensitivity analysis, we obtain the estimate of the VI-MIDAS model parameters $\boldsymbol{\ell}$. The model fit is numerically evaluated using the posterior predictive check [Rubin, 1984, Gelman et al., 2013] on the full data. The procedure requires generating $m = 100$ posterior samples, denoted by the random variable $\mathbf{W}^{rep} = [w_{ij}^{rep}] \in \mathbb{R}_+^{n \times q}$, and then computing the p-value of the model fit as

$$\text{p-value} = p(t(\mathbf{W}^{rep}) < t(\mathbf{W})),$$

where t is the test statistic. In practice, we use the test statistics $t(\mathbf{W}^{rep}) = \mathbf{E}(\log p(\mathbf{W}^{rep}|\boldsymbol{\ell}))$ and $t(\mathbf{W}) = \mathbf{E}(\log p(\mathbf{W}|\boldsymbol{\ell}))$. For the selected setting, we have p-value = 0.53, and thus we fail to reject the hypothesis that the posterior samples are different from the fitted $\mathbf{W}$. A simpler test statistic is $t(\mathbf{w}) = \mathbf{w}$. In this case too, we have p-value = 0.59. We visually examine the model fit by comparing the abundance data with its predicted value and the error; see Figure S8(a-c). Figure S8 (d) reports the convergence of the ELBO using ADVI. Finally, we compare the observed and estimated abundance profiles of the species using the Q-Q plot (for distribution) and the scatter plot; see Figure S8(e-f).

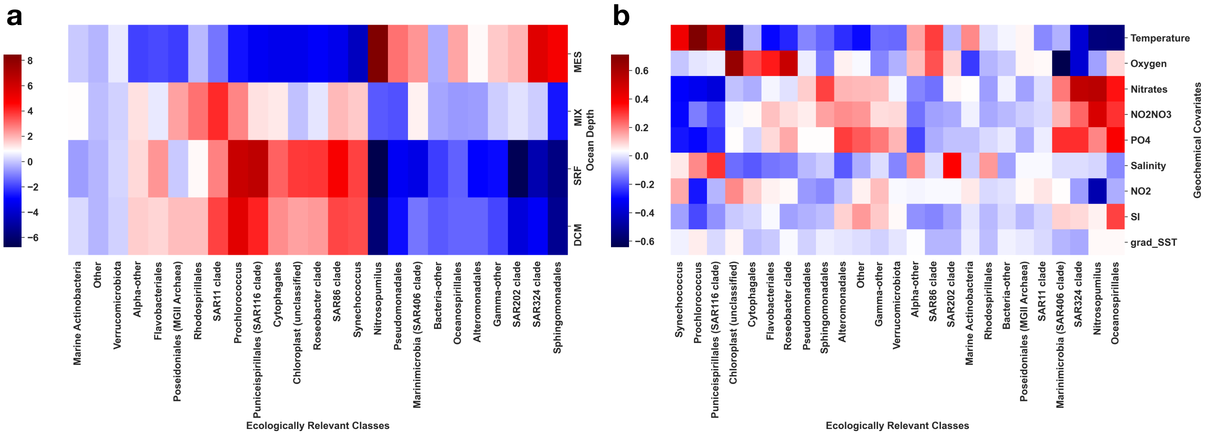

Figure S8: Model fit diagnostic: a) Heatmap showing the abundance profile $\log(\mathbf{W}+1)$ of 1378 species at $n = 139$ distinct geographical locations across the globe; b) Expected value of the abundance using the hyperparameter corresponding to best model fit; c) Error plot representing the absolute value of the difference between the observed and expected species abundance, denoted by $|\log \mathrm{E}(W) - \log \mathbf{W}|$; d) Convergence of the ELBO with conformable rank $k = 16$ and hyperparameter $\lambda = 398.199$ and $\upsilon = 0.04904$; e) Q-Q plot comparing the observed and estimated abundance profile of the species; f) Scatter plot comparing entries of the CLR transform of the observed and estimated microbial species abundance, given by $\log \mathbf{W}/\mathbf{T}$ and $\log \mathrm{E}(\mathbf{W})/\mathbf{T}$, respectively; g) Histogram comparing the distribution of the observed and expected species abundance in terms of $\log \mathbf{W}$ and $\log \mathrm{E}(\mathbf{W})$.

4 Network analysis of mutualistic and competitive interaction

In the model inference, we have considered the top five positive and top five negative entries in the rows of $\mathbf{I}$ to learn about each taxa' most significant mutualistic and competitive interactions. Based on the significant entries in $\mathbf{I}$, we separately define the adjacency matrix of the mutualistic (positive entries) and competitive (negative entries) interactions and then visualize them through the network plots; see Figure S9 (a-b).

We have also identified the most important OTUs/taxa in the interaction networks (based on adjacency matrix; see Figure S9 (a-b)) as a hub (denoted with a "*" shape) with a degree greater than one hundred. The associations of each of the hub nodes with other taxa (in term ERC types) are summarized in the mutualistic and competitive interaction heatmap in the Figure S9 (c). Each of the entries in the heatmap reports the fraction of an ERC type (x-axis) associated with a hub node (y-axis). We have further summarized the most important associations (greater than 0.5 in Figure S9) of hub nodes in the mutualistic and competitive interactions network in Table S5.

The set of OTUs (out of a total of 1378) that exhibit the most significant mutualistic interaction includes {OTU2, OTU3, OTU4, OTU11, OTU13, OTU18, OTU25, OTU21, OTU32, OTU36, OTU48, OTU90}; see Table S5 for the details of taxonomic rank. The ECR types of their hub nodes mainly comprise SAR11 clade, Synechococcus, Prochlorococcus, Rhodospirillales, and Planctomycetes. Based on the ERC type of the nodes connected to OTU2, OTU4, OTU25 and OTU90 (see Table S5), we deduce the existence of subtypes of Rhodospirillales that may differ in ecology and metabolic functions. Using organic substrates as the carbon source, Rhodospirillales can grow in a variety of conditions such as a) anaerobically in the light, b) aerobically in the dark, and c) fermentatively (anaerobically) in the dark [Alber, 2009]. Similarly, based on the nodes connected to OTU11, OTU13, OTU18, OTU22 and OTU32, one can learn about the metabolic versatility of the most abundant ERC type SAR11 clade; and its ability to exhibit mutualistic

relationships with other species.

Now, the set of OTUs that exhibit the most significant competitive interactions includes {OTU19, OTU37, OTU354, OTU1139, OTU1563, OTU1824, OTU2578, OTU5312, OTU4327, OTU4683, OTU4704}; see Table S5 for the details of taxonomic rank. In competitive interactions, one species may discourage the abundance of the other either directly (gazing) or indirectly (metabolic pathway). Most of the OTU that act as hub nodes have very low mean abundance except {OTU19, OTU37, OTU354}. Based on the competitive interactions of OTU19, the analysis suggests that SAR86 clade discourages the abundance of taxa with the ERC indicators Bacteria-other, Oceanospirillales, SAR202 clade, SAR324 clade, Pseudomonadales, Nitrosopumilus, and Sphingomonadales. Similarly, OTU19, a Gamma-other bacteria, reduces the abundance of Poseidoniales (MGII Archaea) and Prochlorococcus, and OTU354, a Marine Actinobacteria, reduces the abundance of Pseudomonadales and Sphingomonadales.

Table S5: Description of the hub nodes in mutualistic and competitive interactions network (see Figure S9 (a-b) of the SM) in terms of their ID, ERC indicator, and mean abundance and their most significant associations (greater than 0.5 in Figure S9(c) of the SM) with other ERC types.

Significant Interaction Type	HUB Marker	OTU ID	HUB ERC	Abundance (Mean)	Connection ERC
	4	OTU2	Rhodospirillales	227.3	Rhodospirillales, Alpha-other, SAR86 clade, SAR11 clade, Synechococcus, Puniceispirillales (SAR116 clade), Prochlorococcus
	5	OTU3	Prochlorococcus	193.7	Poseidoniales (MGII Archaea), SAR86 clade, Synechococcus, Prochlorococcus, Puniceispirillales (SAR116 clade)
	12	OTU4	Rhodospirillales	177.7	Gamma-other, SAR202 clade, Marinimicrobia (SAR406 clade), SAR324 clade, Nitrosopumilus
	2	OTU11	SAR11 clade	99.1	SAR11 clade
	11	OTU13	SAR11 clade	97.7	SAR202 clade
	1	OTU18	SAR11 clade	90.0	Rhodospirillales, SAR86 clade, Synechococcus, SAR11 clade, Prochlorococcus, Puniceispirillales (SAR116 clade)
**Mutualistic**	0	OTU25	Rhodospirillales	86.5	Poseidoniales (MGII Archaea), SAR202 clade, Synechococcus, Prochlorococcus
	14	OTU21	SAR11 clade	85.2	Nitrosopumilus, SAR324 clade
	3	OTU32	SAR11 clade	73.1	Poseidoniales (MGII Archaea)
	10	OTU36	SAR86 clade	73.0	Puniceispirillales (SAR116 clade)
	13	OTU48	Planctomycetota	72.0	SAR202 clade, SAR324 clade
	6	OTU90	Rhodospirillales	51.2	Alteromonadales, Gamma-other, Pseudomonadales, SAR202 clade
	16	OTU19	SAR86 clade	80.7	Bacteria-other, Oceanospirillales, SAR202 clade, SAR324 clade, Pseudomonadales, Nitrosopumilus, Sphingomonadales
	2	OTU37	Gamma-other	71.3	Poseidoniales (MGII Archaea), Prochlorococcus
	9	OTU354	Marine Actinobacteria	22.1	Pseudomonadales, Sphingomonadales
	15	OTU1139	Flavobacteriales	9.0	Alteromonadales, Oceanospirillales, SAR202 clade, SAR324 clade, Nitrosopumilus, Pseudomonadales, Sphingomonadales
	1	OTU1563	Flavobacteriales	8.2	Poseidoniales (MGII Archaea), SAR11 clade, SAR86 clade, Synechococcus, Prochlorococcus
**Competitive**	0	OTU1824	Oceanospirillales	6.9	Alpha-other, Marine Actinobacteria, Rhodospirillales, Chloroplast (unclassified), Cytophagales, Puniceispirillales (SAR116 clade), SAR11 clade, SAR86 clade, Poseidoniales (MGII Archaea), Synechococcus, Prochlorococcus
	13	OTU2578	Marine Actinobacteria	6.0	SAR202 clade, SAR324 clade, Nitrosopumilus, Pseudomonadales, Sphingomonadales
	3	OTU5312	Chloroplast (unclassified)	4.6	Prochlorococcus
	11	OTU4327	Cytophagales	4.6	Bacteria-other, Roseobacter clade
	4	OTU4683	Alteromonadales	4.5	Prochlorococcus, Chloroplast (unclassified), Puniceispirillales (SAR116 clade), Synechococcus
	5	OTU4704	Alteromonadales	4.5	SAR202 clade, SAR324 clade, Nitrosopumilus, Sphingomonadales, Pseudomonadales

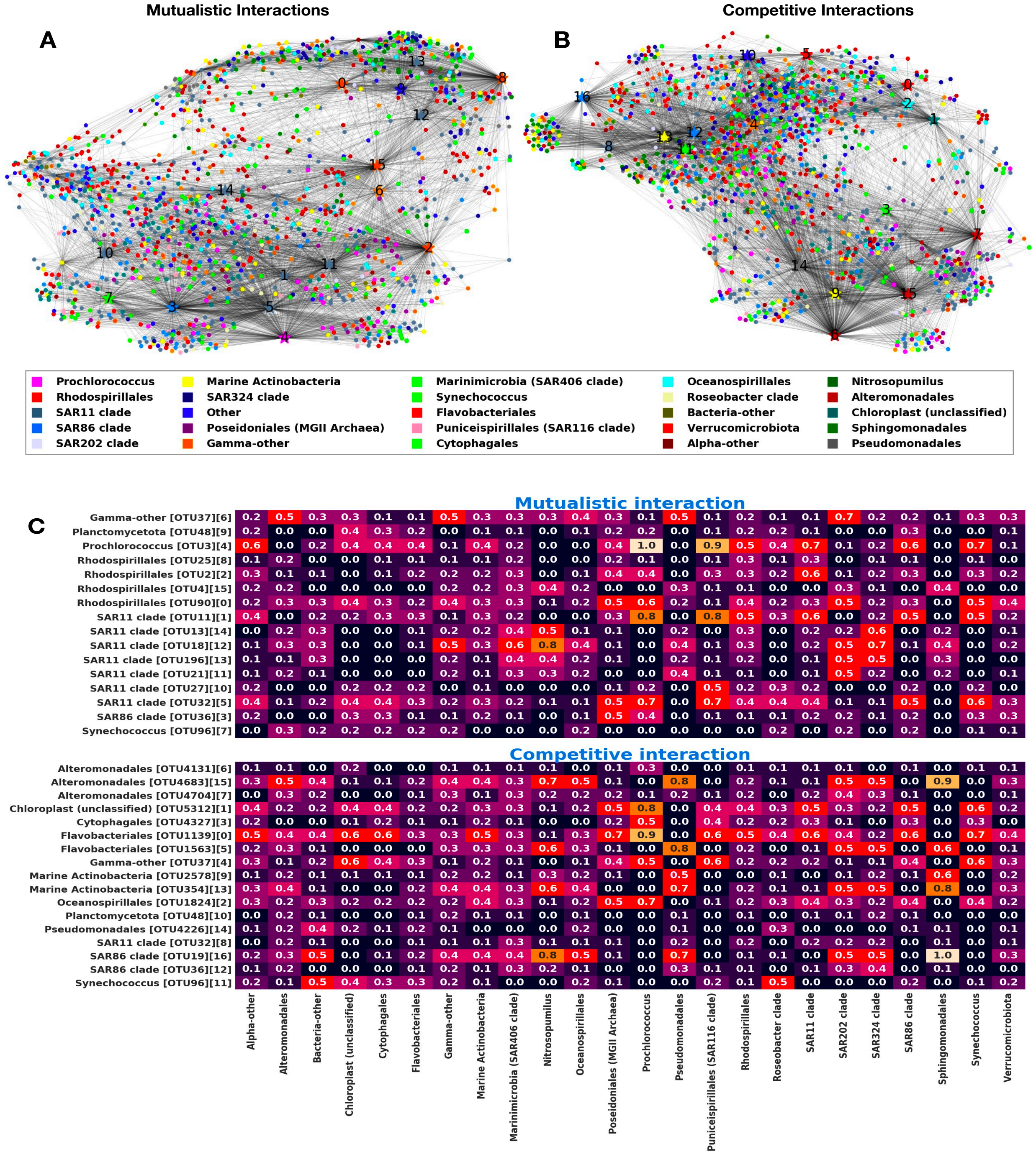

Figure S9: Interactions among species: Based on the significant entries in **I**, we separately define the adjacency matrix of the mutualistic (positive entries) and competitive (negative entries) interactions and then visualize them through the network plots. a) Network plot based on the top 5 positive interactions of each of the OTUs with "*" denoting the ten hub nodes identified with degree greater than 100. b) Network plot based on the top 5 negative interactions of each of the OTUs with "*" representing the 11 hub nodes identified with degree greater than 100. c) Distribution of the association of the hub nodes (y-axis) with other species. Entries in the heatmap show association of a hub node with the fraction of each of the ERC types (x-axis).

5 Comparison of VI-MIDAS with existing framework

5.1 Comparison of VI-MIDAS network with microbial co-occurance network

We explored microbial community relationships by conducting a co-occurrence network analysis and comparing it to a network inferred using the VI-MIDAS framework. For the co-occurrence network, we used log-transformed abundance data and employed Spearman's rank correlation ($|r| > 0.3$) to measure associations between microbial taxa. To refine the network structure and improve interpretability, we applied a k -nearest neighbor algorithm (k = 10), ensuring that each node connected only to its ten most correlated taxa. Visualizing the network with a force-directed graph layout and identified seven distinct ecological modules using Clauset-Newman-Moore modularity optimization algorthm (see Figure S10). We have summarizes top five or less number of taxa in each of the seven modules identified in Table S6. *These modules highlighted groups of taxa with closely linked ecological interactions based on their occurrences.* In contrast, the VI-MIDAS framework took a more integrative approach by combining microbiome data with environmental and spatiotemporal covariates. The method provides framework to account for confounding biogeochemical factors such as depth, salinity, and sea surface temperature. The resulting network identified five ecological modules, reflecting taxa with shared environmental dependencies. Notably, Alteromonadales-dominated Modules 4 and 5 in the VI-MIDAS network demonstrated distinct environmental preferences, aligning with their known ecological roles.

Table S7 summarizes the significant structural differences we found between the two networks. The two networks' significant differences in edge connectivity were indicated by a Hamming distance of 0.915. The co-occurrence network displayed a higher clustering coefficient (0.441 vs. 0.400), suggesting tighter taxonomic groups, whereas the VI-MIDAS network displayed a slightly higher density (0.0116 vs. 0.0111). The VI-MIDAS network has a reduced average shortest path length (4.732 vs. 5.180), indicating more effective node connectivity. While highlighting the distinctive features that each method captures, similarity metrics like the Adjusted Rand Index (ARI) and Normalized Mutual Information (NMI) revealed a moderate overlap in community structures Rawlings et al. [2023]. As indicated in Table S8, we conducted statistical tests comparing important network features in order to investigate the differences in more detail. These tests revealed significant differences in degree centrality ($p = 4.6 \times 10^{-7}$), closeness centrality ($p = 7.9 \times 10^{-127}$), and spectral correlation ($p = 6.2 \times 10^{-12}$), reflecting distinct patterns of node influence and global connectivity.

Through this analysis, we gained a deeper understanding of how the two approaches capture different aspects of microbial ecology. The co-occurrence network emphasized direct microbial interactions, while VI-MIDAS provided a broader perspective by integrating spatiotemporal and environmental dependencies.

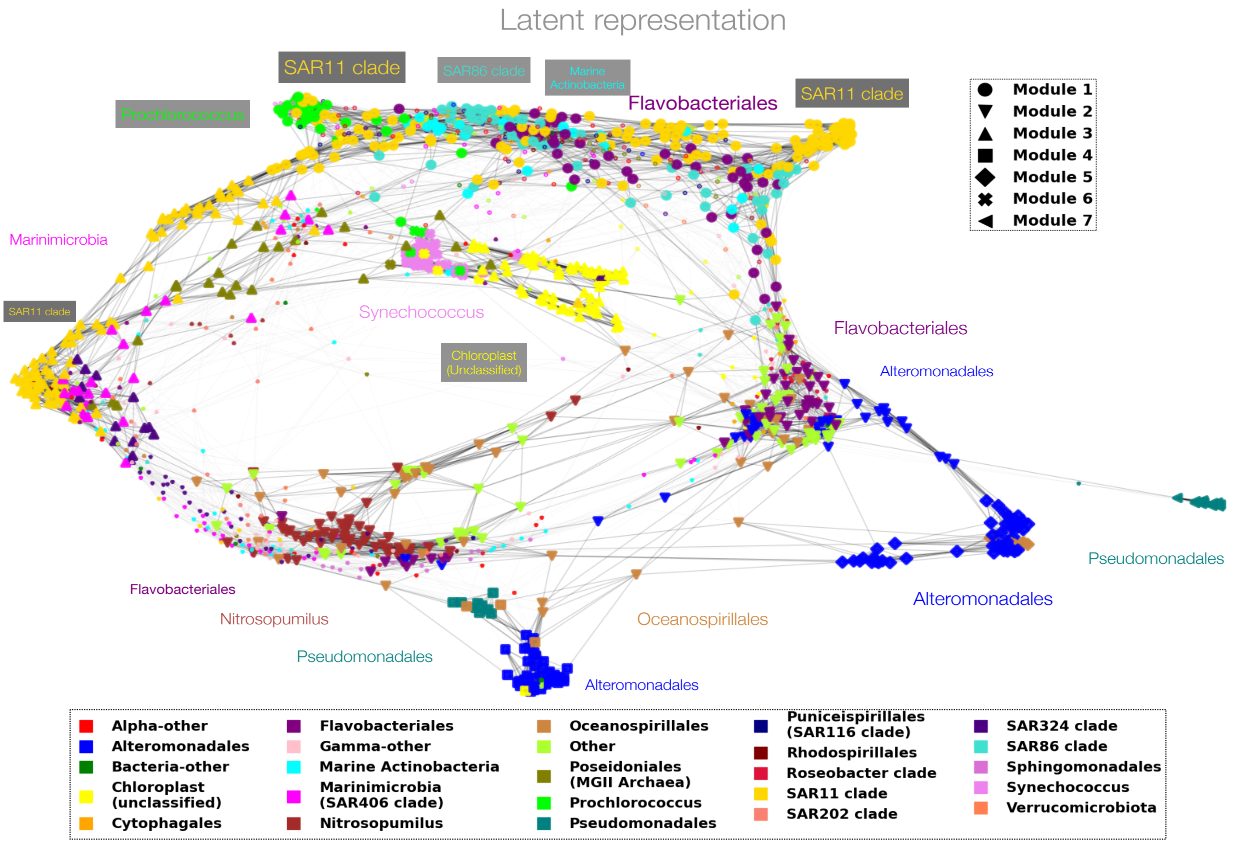

Figure S10: Network visualization of microbial taxa co-occurrence based on log-transformed abundance data, constructed using Spearman's correlation and refined with the k -nearest neighbor algorithm ($k = 10$). Nodes represent taxa, and edges highlight significant associations, with modular structures indicating ecological interactions and shared functional roles.

Alteromonadales dominated subnetwork: We further analyze and compare the Alteromonadales-dominated modules from the VI-MIDAS framework (M4-M5) to that of sub-network based on microbial co-occurrence. Table S9 presents statistical test results to demonstrate significant differences between them in terms of various network properties. For instance, in terms of closeness centrality and spectral correlation the two networks are significantly different, indicating distinct global connectivity and structural patterns between the two networks.

Figure S11 compare the two subnetwork in terms phylogenetic distribution analysis. In case of VI-MIDAS, Pseudoalteromonas and Rheinheimera are mainly dominant in Module 4, whereas Alteromonas, Salinimonas, Idiomarina, and Glaciecola is present in Module 5. This demonstrate the framework's ability to capture functional and ecological differences within this taxon. By accounting for environmental and spatio-temporal factors, VI-MIDAS identifies functionally similar species that share ecological niches. The distinction between these subgroups reflects adaptations to specific environmental conditions, highlighting how VI-MIDAS integrates ecological context to reveal taxonomically related and functionally similar microbial groups.

Together, these methodologies enhance our understanding of microbial dynamics by combining strengths: VI-MIDAS excels in integrating environmental and latent factors to capture indirect dependencies, while the co-occurrence network highlights direct taxon-taxon interactions, making it useful for analyzing immediate

Table S6: Co-occurance network analysis: Top five ecologically relevant classifications (ERC) indicator of taxa in the seven modules identified in the network (shown in Figure S11) highlighting similar nodes. The table reports the composition (as %) of 825 taxa in terms of the ERC indicator and their mean abundance.

Module	ERC	Abundance (Mean)	Members (%)	Module	ERC	Abundance (Mean)	Members (%)
**1**	SAR11 clade	38.68	10.23	**4**	Alteromonadales	15.19	3.05
**1**	SAR86 clade	27.47	3.92	**4**	Pseudomonadales	9.54	0.65
**1**	Flavobacteriales	19.54	3.34	**4**	Oceanospirillales	18	0.22
**1**	Prochlorococcus	53.73	2.03	**4**	Other	13.29	0.07
**1**	Marine Actinobacteria	25.48	1.60	**4**	Chloroplast (unclassified)	17.81	0.07
**2**	Other	9.86	4.14	**5**	Alteromonadales	7.7	3.19
**2**	Nitrosopumilus	19.82	3.63	**5**	Oceanospirillales	9.25	0.44
**2**	Flavobacteriales	8.08	3.12	**6**	Synechococcus	19.91	2.54
**2**	Oceanospirillales	9.13	3.05	**6**	Prochlorococcus	15.54	0.29
**2**	Alteromonadales	5.39	2.32	**6**	Chloroplast (unclassified)	19.95	0.22
**3**	SAR11 clade	22.11	6.02	**6**	Poseidoniales (MGII Archaea)	10.58	0.07
**3**	Chloroplast (unclassified)	14.62	4.43	**6**	Marine Actinobacteria	12.97	0.07
**3**	Poseidoniales (MGII Archaea)	23.98	1.74	**7**	Pseudomonadales	5.06	1.02
**3**	Marinimicrobia (SAR406 clade)	21.24	1.74				
**3**	SAR324 clade	19.22	1.09				

Table S7: Comparison of VI-MIDAS and microbial co-occurrence network analysis approaches based on various network measures.

Measure	VI-MIDAS	Co-occurrence	Dissimilarity
Hamming Distance			0.915
Network Density	0.0116	0.0111	0.001
Network Clustering Coefficient	0.400	0.441	-0.041
Network Average Shortest Path Length	4.732	5.180	-0.448
Adjusted Rand Index			0.212
Normalized Mutual Information			0.309

Table S8: Statistical test comparing VI-MIDAS and microbial co-occurrence network for some of the key network properties . These metrics assess the structural and functional similarities between networks, with lower p-values indicating statistically significant differences.

Measure	Test Statistic	P-value
Degree Distribution	0.105225	1.052250e-01
Degree Centrality	0.105225	4.622368e-07
Betweenness Centrality	0.058055	1.921257e-02
Closeness Centrality	0.451379	7.922546e-127
Spectral Correlation	0.183795	6.190849e-12

microbial relationships. These complementary insights not only improve our ability to study microbial communities but also provide a robust framework for advancing microbiome research in complex ecosystems.

Figure S11 shows assoiciation Alteramonadales in M4-M5 with environmental factors. The associations of microbial taxa with oxygen and nitrate concentrations shows their ecological adaptations and metabolic strategies in oceanic environments. Pseudoalteromonas and Rheinheimera, positively associated with oxygen, thrive in oxic conditions, likely contributing to organic matter degradation and secondary metabolite production in oxygen-rich zones [Møller et al., 2022, Long et al., 2021, Li et al., 2024]. Conversely, Alteromonas, Salinimonas, Idiomarina, and Glaciecola, associated with low oxygen but high nitrate levels, are adapted to oxygen minimum zones (OMZs) where nitrate serves as a key electron acceptor for denitrification

or nitrate reduction [Long et al., 2021, Acinas et al., 2021]. These patterns highlight oxygen and nitrate as critical drivers of microbial community structure, shaping ecological niches and supporting biogeochemical processes essential for maintaining ocean ecosystem balance [Møller et al., 2022, Long et al., 2021, Li et al., 2024].

Table S9: Sub-network associated with Alteramonadales: Statistical test result comparing key network properties of VI-MIDAS and microbial co-occurrence network. Except ARI and NMI, these metrics assess the structural and functional similarities between networks, with lower p-values indicating statistically significant differences.

Measure	Test Statistic/ Value	Signifigance (P-value)
Adjusted Rand Index (ARI)	0.368678	
Normalized Mutual Information (NMI)	0.308652	
Degree Centrality	0.100000	0.587976
Betweenness Centrality	0.091667	0.69668
Closeness Centrality	0.708333	3.19e-29
Spectral Correlation	0.377398	6.19e-12

5.2 Comparison with linear regression model

Here we performed a comparative analysis by fitting a sparse linear regression model to the normalized microbiome data for each OTU, using environmental features and spatiotemporal variables as predictors (See Figure S12), and compares the associations of OTUs in the five modules identified by VI-MIDAS with those obtained using sparse linear regression. While the linear regression approach identified some general trends, it failed to capture the latent, higher-order interactions and nuanced community structures that VI-MIDAS uncovers. Furthermore, the ablation study (Results, page 7, paragraph 4, and Supplementary Table S4) confirmed the importance of each component in VI-MIDAS, demonstrating that removing taxon-taxon interaction terms significantly degrades model performance.

5.3 Comparison with variance partition analysis

Variation partitioning analysis (VPA) is a commonly used approach in microbial ecology to partition the variation in microbial community composition based on environmental variables. The analysis thus provide a broad understanding of how abiotic factors, such as temperature, salinity, and nutrient concentrations, contribute to microbial abundances at the community level. However, it is limited when the interest lies in partitioning variation at the level of individual taxa or specific ecological response categories (ERCs). In addition, the approach does not integrate latent taxon-taxon interactions or explicitly model the joint effects of abiotic and biotic drivers, which are critical for a comprehensive understanding of microbial ecology.

In contrast, VI-MIDAS offers a probabilistic generative model that not only captures non-linear relationships between microbial abundances and environmental variables (e.g., depth, temperature, spatiotemporal features) but also integrates latent taxon-taxon interactions. This approach enables partitioning variation at both the community and individual taxa or ERC levels. For example, VI-MIDAS identifies ecologically relevant microbial modules, such as SAR11 and Nitrosopumilus, and reveals hidden taxon relationships that cannot be resolved by traditional VPA or distance-based methods. Furthermore, it uncovers latent microbial sub-communities and their biogeographic patterns, providing deeper ecological insights (*Main manuscript, Results, page 7, Fig. 5*).

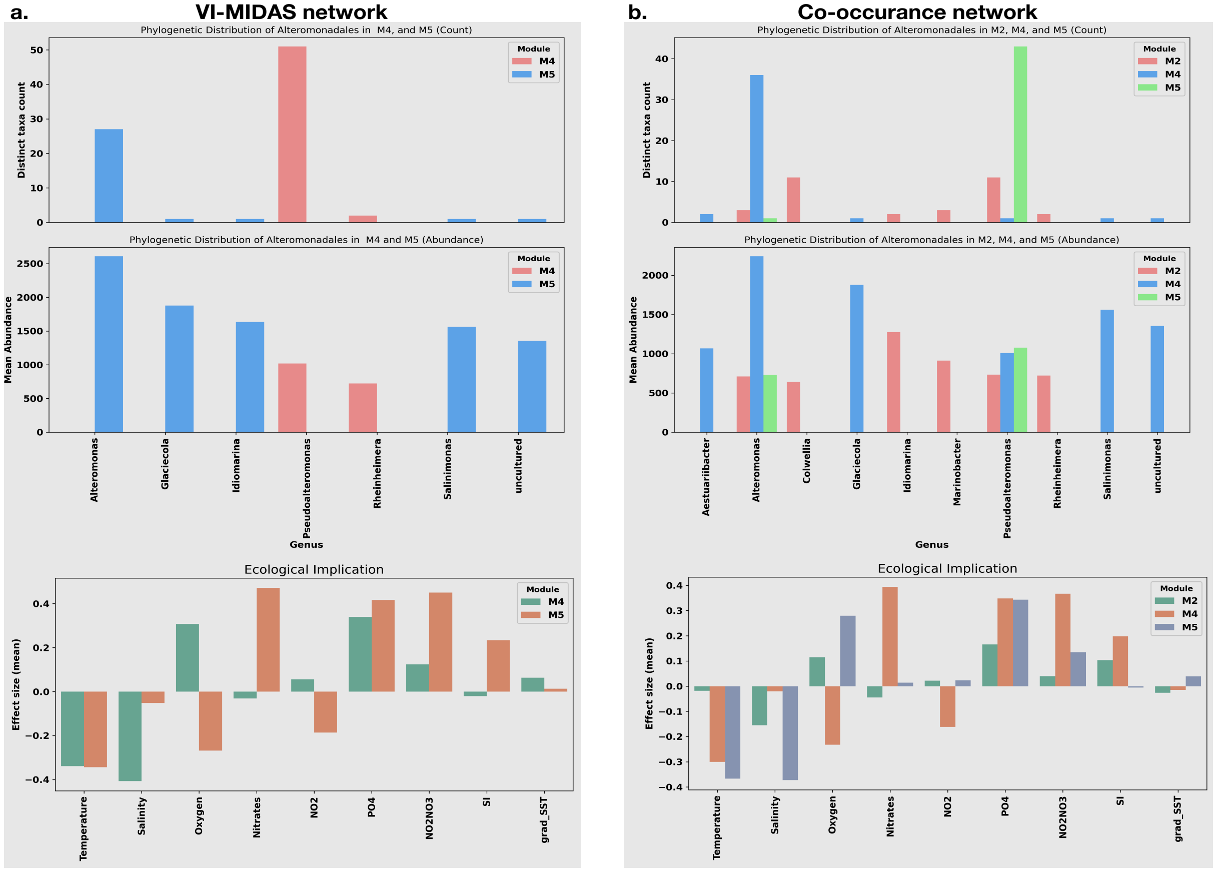

Figure S11: Comparison of Alteromonadales subnetworks in modules identified by VI-MIDAS and microbial co-occurrence networks. The first subplot (VI-MIDAS) shows the phylogenetic distribution of distinct taxa in Module 4 and Module 5, reported as counts and mean abundances at the genus level. The second subplot (co-occurrence network) reports the same for Alteromonadales in Module 2, Module 4, and Module 5.

References

Silvia G Acinas, Pablo Sánchez, Guillem Salazar, Francisco M Cornejo-Castillo, Marta Sebastián, Ramiro Logares, Marta Royo-Llonch, Lucas Paoli, Shinichi Sunagawa, Pascal Hingamp, et al. Deep ocean metagenomes provide insight into the metabolic architecture of bathypelagic microbial communities. *Communications biology*, 4(1):604, 2021.

B.E. Alber. Autotrophic co2 metabolism. In Moselio Schaechter, editor, *Encyclopedia of Microbiology (Third Edition)*, pages 18–31. Academic Press, Oxford, third edition edition, 2009. ISBN 978-0-12-373944-5. doi: https://doi.org/10.1016/B978-012373944-5.00064-X. URL `https://www.sciencedirect.com/science/article/pii/B978012373944500064X`.

David M Blei, Alp Kucukelbir, and Jon D McAuliffe. Variational inference: A review for statisticians. *Journal of the American statistical Association*, 112(518):859–877, 2017.

Ben J Callahan, Kris Sankaran, Julia A Fukuyama, Paul J McMurdie, and Susan P Holmes. Bioconductor workflow for microbiome data analysis: from raw reads to community analyses. *F1000Research*, 5, 2016.

Bob Carpenter, Andrew Gelman, Matthew D Hoffman, Daniel Lee, Ben Goodrich, Michael Betancourt,

Marcus Brubaker, Jiqiang Guo, Peter Li, and Allen Riddell. Stan: A probabilistic programming language. *Journal of statistical software*, 76(1), 2017.

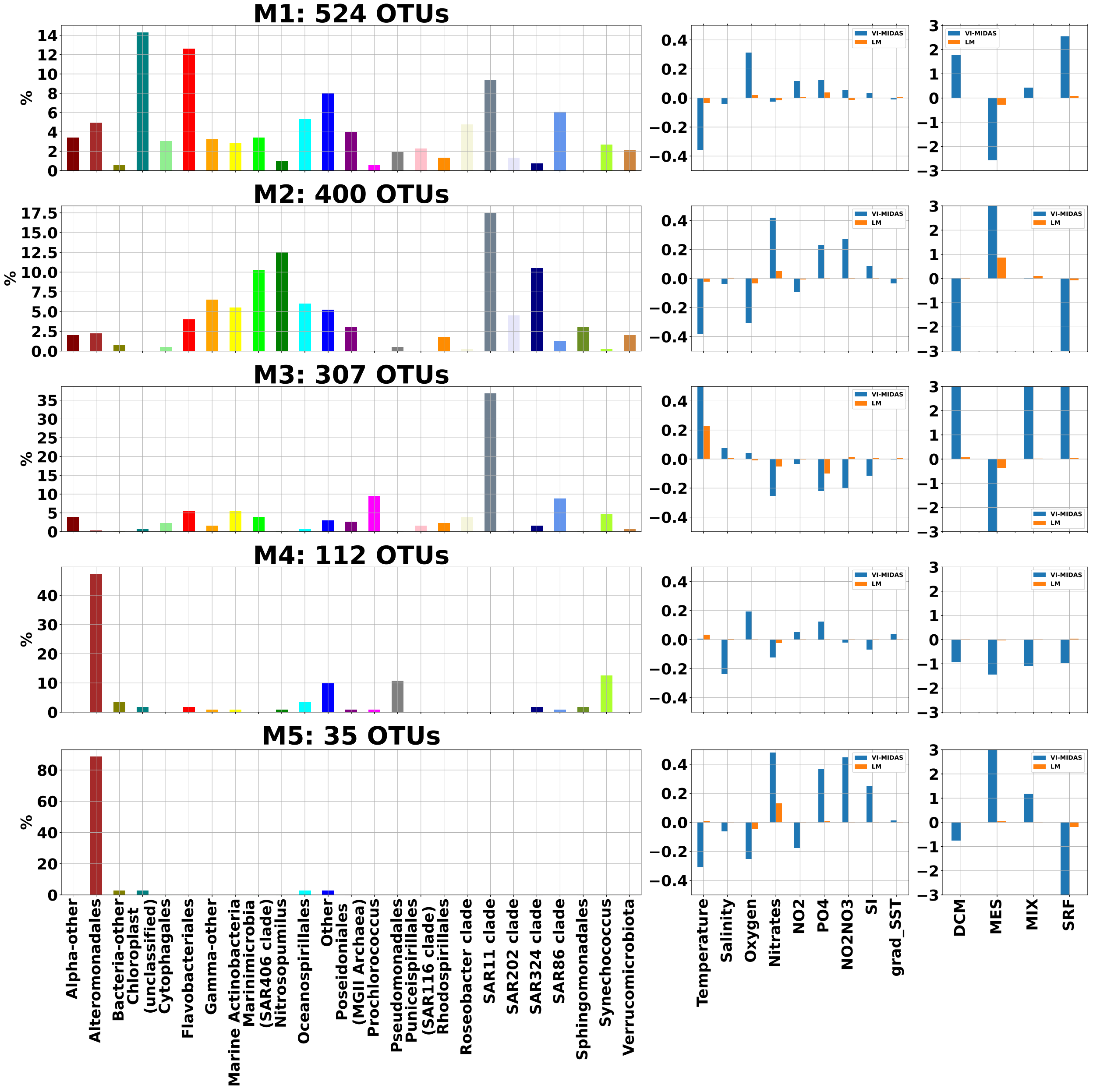

Figure S12: Global associations of biogeography and covariates: Each row presents the average effect size of the association between the microbial abundances of taxa in a module (M1-M5) to the geochemical features and ocean depth (from left to right). A module (leftmost) is shown as the composition (in %) of the ERCs. Each module comprises different number of taxa {524, 400, 307, 112, 35}, respectively. Modules M1-M3 cover the majority of taxa, and M4-M5 two smaller Alteromonadales-dominated sub-communities.Rightmost two panel show comparison of the association of biogeography and covariates using VI-MIDAS and linear regression model.

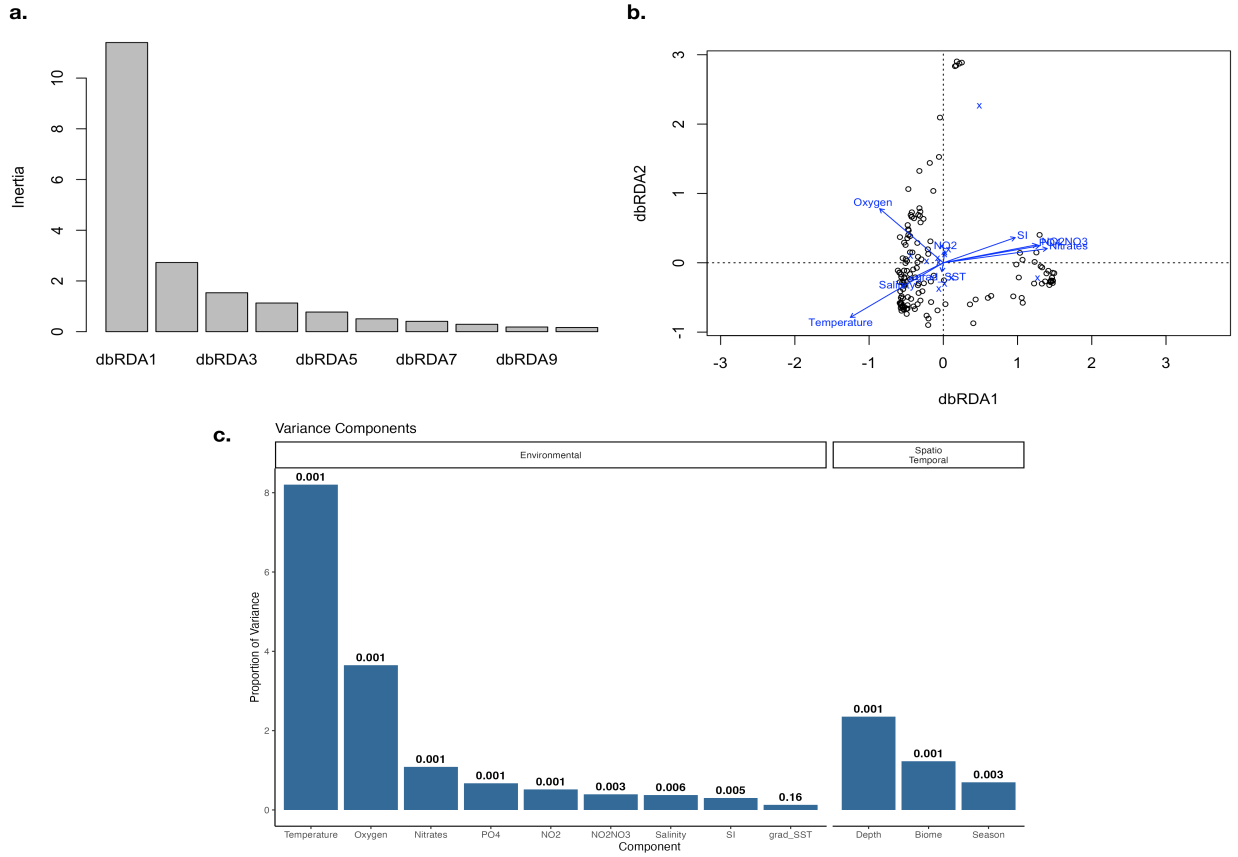

Figure S13: Variation partition analysis of microbiome data from the Tara Ocean Expedition. (a) Scree plot showing the proportion of variation explained by environmental, spatial, and temporal factors. (b) Biplot illustrating sample distribution and environmental predictors. (c) PERMANOVA analysis highlighting the significance and relative contributions of spatial, temporal, and environmental factors to microbial community variation.

Roberto de la Cruz and Jan-Ulrich Kreft. Geometric mean extension for data sets with zeros. *arXiv preprint arXiv:1806.06403*, 2018.

John Duchi, Elad Hazan, and Yoram Singer. Adaptive subgradient methods for online learning and stochastic optimization. *Journal of machine learning research*, 12(Jul):2121–2159, 2011.

AB Gelman, JB Carlin, HS Stern, DB Dunson, A Vehtari, and D Rubin. Bayesian data analysis third edition. boca raton. *FL: CRC Press.[Google Scholar]*, 2013.

Michael I Jordan, Zoubin Ghahramani, Tommi S Jaakkola, and Lawrence K Saul. An introduction to variational methods for graphical models. *Machine learning*, 37(2):183–233, 1999.

Alp Kucukelbir, Dustin Tran, Rajesh Ranganath, Andrew Gelman, and David M Blei. Automatic differentiation variational inference. *The Journal of Machine Learning Research*, 18(1):430–474, 2017.

Jiaqian Li, Ningdong Xie, Xiuping Liu, Mohan Bai, Dana E Hunt, and Guangyi Wang. Oxygen levels differentially attenuate the structure and diversity of microbial communities in the oceanic oxygen minimal zones. *Science of The Total Environment*, 948:174934, 2024.

Andrew M Long, Sophie K Jurgensen, Ariel R Petchel, Emily R Savoie, and Jennifer R Brum. Microbial ecology of oxygen minimum zones amidst ocean deoxygenation. *Frontiers in Microbiology*, 12:748961, 2021.

Aditya Mishra et al. Robust regression with compositional covariates. *arXiv preprint arXiv:1909.04990*, 2019.

Tor Einar Møller, Sven Le Moine Bauer, Bjarte Hannisdal, Rui Zhao, Tamara Baumberger, Desiree L Roerdink, Amandine Dupuis, Ingunn H Thorseth, Rolf Birger Pedersen, and Steffen Leth Jørgensen. Mapping microbial abundance and prevalence to changing oxygen concentration in deep-sea sediments using machine learning and differential abundance. *Frontiers in Microbiology*, 13:804575, 2022.

Craig M. Rawlings, Jeffrey A. Smith, and Daniel A. McFarland. *Network Analysis: Integrating Social Network Theory, Method, and Application with R*, volume 52 of *Structural Analysis in the Social Sciences*. Cambridge University Press, Cambridge, 2023. ISBN 9781009272288.

Donald B Rubin. Bayesianly justifiable and relevant frequency calculations for the applied statistician. *The Annals of Statistics*, pages 1151–1172, 1984.

Pixu Shi, Anru Zhang, Hongzhe Li, and Others. Regression analysis for microbiome compositional data. *Ann. Appl. Stat.*, 10(2):1019–1040, 2016.

Martin J Wainwright, Michael I Jordan, et al. Graphical models, exponential families, and variational inference. *Foundations and Trends® in Machine Learning*, 1(1–2):1–305, 2008.

Xinyan Zhang, Himel Mallick, Zaixiang Tang, Lei Zhang, Xiangqin Cui, Andrew K Benson, and Nengjun Yi. Negative binomial mixed models for analyzing microbiome count data. *BMC bioinformatics*, 18(1):4, 2017.
